# Supplementary material for: A Comprehensive Assessment of Ultraviolet-Radiation-Induced Mutations in Flammulina filiformis Using Whole-Genome Resequencing
Source: J Fungi (Basel). 2024 Mar 20;10(3):228. doi: 10.3390/jof10030228 (PMC10971301; doi:10.3390/jof10030228)
Supplement: Supplementary file 1 [file jof-10-00228-s001.zip › Supplementary Material S8/KEGG annotation/out/64381550635650.os/KO/out_map/map05340.html]

KEGG PATHWAY: Primary immunodeficiency - Reference pathway


|  |  |
| --- | --- |
| **Primary immunodeficiency - Reference pathway** |  |

[
Pathway menu
| Organism menu
| Pathway entry
| Show description
| User data mapping
]

Primary immunodeficiencies (PIs) are a heterogeneous group of disorders, which affect cellular and humoral immunity or non-specific host defense mechanisms mediated by complement proteins, and cells such as phagocytes and natural killer (NK) cells. These disorders of the immune system cause increased susceptibility to infection, autoimmune disease, and malignancy. Most of PIs are due to genetic defects that affect cell maturation or function at different levels during hematopoiesis. Disruption of the cellular immunity is observed in patients with defects in T cells or both T and B cells. These cellular immunodeficiencies comprise 20% of all PIs. Disorders of humoral immunity affect B-cell differentiation and antibody production. They account for 70% of all PIs.

|  |  |
| --- | --- |
| Reference pathway | 100% |
